# Twenty Years of Schizophrenia Research in the Northern Finland Birth Cohort 1966: A Systematic Review

**DOI:** 10.1155/2015/524875

**Published:** 2015-05-18

**Authors:** Erika Jääskeläinen, Marianne Haapea, Nina Rautio, Pauliina Juola, Matti Penttilä, Tanja Nordström, Ina Rissanen, Anja Husa, Emmi Keskinen, Riikka Marttila, Svetlana Filatova, Tiina-Mari Paaso, Jenni Koivukangas, Kristiina Moilanen, Matti Isohanni, Jouko Miettunen

**Affiliations:** ^1^Center for Life Course Epidemiology and Systems Medicine, University of Oulu, P.O. Box 5000, 90014 Oulu, Finland; ^2^Medical Research Center Oulu, Oulu University Hospital and University of Oulu, P.O. Box 5000, 90014 Oulu, Finland; ^3^Department of Psychiatry, Center for Clinical Neurosciences, University of Oulu, P.O. Box 5000, 90014 Oulu, Finland; ^4^Department of Psychiatry, Oulu University Hospital, P.O. Box 26, 90029 Oulu, Finland; ^5^Unit of Primary Health Care, Oulu University Hospital, P.O. Box 10, 90029 Oulu, Finland; ^6^Aurora Doctoral Programme, Thule Institute, University of Oulu, P.O. Box 7300, Oulu, Finland

## Abstract

Birth cohort designs are useful in studying adult disease trajectories and outcomes, such as schizophrenia. We review the schizophrenia research performed in the Northern Finland Birth Cohort 1966 (NFBC 1966), which includes 10,934 individuals living in Finland at 16 years of age who have been monitored since each mother's mid-pregnancy. By the age of 44, 150 (1.4%) had developed schizophrenia. There are 77 original papers on schizophrenia published from the NFBC 1966. The early studies have found various risk factors for schizophrenia, especially related to pregnancy and perinatal phase. Psychiatric and somatic outcomes were heterogeneous, but relatively poor. Mortality in schizophrenia is high, especially due to suicides. Several early predictors of outcomes have also been found. Individuals with schizophrenia have alterations in brain morphometry and neurocognition, and our latest studies have found that the use of high lifetime doses of antipsychotics associated with these changes. The schizophrenia research in the NFBC 1966 has been especially active for 20 years, the prospective study design and long follow-up enabling several clinically and epidemiologically important findings. When compared to other birth cohorts, the research in the NFBC 1966 has offered also unique findings on course and outcome of schizophrenia.

## 1. Introduction 

Birth cohort studies were originally designed to study early health outcomes, but as the earliest birth cohorts started in the 1940s to the 1960s have matured, adult outcomes have also been studied [1]. Welham and colleagues [1] reviewed birth cohort studies on antecedents of schizophrenia, finding 11 birth cohorts from seven countries. The authors concluded that birth cohort studies have provided important insights into how the premorbid developmental trajectory of individuals who develop schizophrenia differs from their peers.

The Northern Finland Birth Cohort 1966 (NFBC 1966) is a birth cohort which has been studied extensively with almost 1000 peer-review publications in various fields of medicine (http://www.oulu.fi/nfbc). The NFBC 1966 was collected during the mid-1960s by paediatrician and Professor of Public Health Paula Rantakallio, the first aim being to analyze perinatal health [2]. In the 1980s, adolescent health and, in the 1990s, adult somatic and psychiatric disorders emerged as the main foci. Schizophrenia research in the NFBC 1966 has been especially active, with the first studies performed in the mid-1990s. The first studies focused on early risk factors, but later on clinical and social outcomes, neurocognition, brain morphometry, somatic illnesses, and genetics have also been studied.

In this paper, we systematically review the schizophrenia research performed in the NFBC 1966.

## 2. Methods

### 2.1. Literature Search

The literature search was completed in PubMed in September 2014. The keywords included “Northern Finland Birth Cohort” and words related to schizophrenia (including schizotypy and psychotic disorders). Additionally, we manually searched for articles from the extensive publication list maintained by the NFBC 1966 (http://www.oulu.fi/nfbc). We found 77 original articles related to schizophrenia. In addition, previous reviews from the NFBC 1966 were utilized in this review.

### 2.2. Sample

The sample was drawn from the NFBC 1966, which is based on 12,068 pregnant women and their 12,058 live-born babies in two northernmost Finnish provinces (Oulu and Lapland), with an expected date of birth during 1966 [2]. The live-births in this study represent 96% of all births in the region. The information has been collected during pregnancy, delivery, childhood, and the follow-up periods at adulthood. The schizophrenia studies are based on 10,934 cohort members who lived in Finland in 1982. The original articles of this literature review are largely based on three data collections: the 31-year follow-up of the cohort members alive in 1997, and two smaller psychiatric follow-up studies. Register data and earlier collected data of the NFBC 1966 have also been utilized in the studies. The overall study design has been approved and is under the review of the Ethical Committee of the Northern Ostrobothnia Hospital District.

The 31-year follow-up of the whole cohort was conducted in 1997. Comprehensive data on somatic and mental health and the use of health services and medications as well as socioeconomic status were collected using a postal questionnaire (*N* = 8,297). A clinical examination of the cohort members living in Northern Finland or Helsinki area included, for instance, a blood sample draw, which has been utilized in genetic studies (*N* = 5,960). Additional questionnaires including, for example, schizotypal traits, were also filled in (*N* = 5,084) [41].

The 34-year follow-up for psychoses was conducted in 1999–2001. Cohort members with a psychotic episode by the end of the year 1997 (cases) were detected using the Finnish Hospital Discharge Register (FHDR), currently known as Care Register for Health Care. The control subjects were selected randomly from the cohort members without a psychotic episode, living in the region of Oulu. The 34-year follow-up consisted of structural magnetic resonance imaging (MRI) of the brain (GE Signa 1.5T scanner), measures of cognitive functioning, psychiatric interviews, and questions related to, for example, the use of antipsychotic medication, social background, and substance use. In the 34-year follow-up, 91 cases with a psychotic disorder (61 with schizophrenia) and 104 controls participated and provided written informed consent [42, 43].

The 43-year follow-up for psychoses was conducted in 2008–2010. In addition to those with regard to whom a psychosis had been detected during the 34-year follow-up, those who had developed a psychosis between the years 1998 and 2008 according to the nationwide registers or self-report were invited to participate. The new control subjects were selected randomly from all the cohort members. The procedure was extended from the 34-year follow-up with functional MRI and more measures of cognitive functioning. Out of the eligible subjects, 107 cases with a psychotic disorder (54 with schizophrenia) and 194 controls participated and provided written informed consent. Of the participants, 54 cases with a psychotic disorder (40 with schizophrenia) and 76 controls participated, both at ages 34 and 43 [44].

### 2.3. Diagnostics

The diagnoses of the participants of the 34-year follow-up were validated in accordance with DSM-III-R [45, 46]. New cases with schizophrenia and other psychoses have been included in the later studies: these cases are detected continuously from several nationwide registers. Due to the more comprehensive set of registers, the later studies include more outpatients than the previous studies.

## 3. Risk Factors

The first schizophrenia studies in the NFBC 1966 focused on risk factors. The main results of the studies are presented in Table 1. Twenty original studies and also some reviews (e.g., [47]) concerning the various risk factors of schizophrenia have been published. These risk factors can be divided into risks during pregnancy and birth, childhood, and adolescence and later premorbid factors.

The most essential risk factors identified in the latest NFBC 1966 review [47] were male gender, parental psychosis, unwanted pregnancy, perinatal brain damage, low birth weight, late age at learning to stand and walk, not being potty-trained by the age of one, central nervous system infections by the age of 14 years, and not attending normal school grade.

Later, other risk factors have also been found, such as severe injuries [19] and both high and low birth length and weight [5, 10]. New aspects of the studies were to link parental psychosis and known risk factors [5, 22, 24] and to study their interactions. It has been found, for instance, that the presence of early biological risk factors increased the risk of schizophrenia only among those with parental psychosis [5] and that low birth weight and length increased the risk only among those without parental psychosis, whereas high birth weight and length did so only among those with parental psychosis [5].

In each stage of human development, there are risk factors influencing the occurrence of schizophrenia. It is important to identify the risk and create awareness among health professionals and decision makers to develop and implement preventive strategies, though it should be noted that most of the effect sizes detected are small or medium level. In schizophrenia risk studies in general, the risk factors with highest quality evidence have shown medium effect sizes [48].

## 4. Course of Illness and Outcomes

### 4.1. Course of Illness and Outcomes

In the NFBC 1966 on average of ten years from the onset of illness, the outcomes were heterogeneous, but relatively poor [25, 49]. A total of 81% of subjects with schizophrenic psychoses needed rehospitalization [26]. Adult educational level was significantly lower in subjects with schizophrenia, compared to the controls [50].

At the age of 34, outcomes were heterogeneous and varied largely, depending on the measures of outcomes. More than half (56%) of the individuals with schizophrenia were on disability pension [34], whereas 21% were in symptomatic remission, with only 3.4% having recovered [25, 49]. These numbers are somewhat smaller than the average rate of recovery (13.5%) worldwide, based on a meta-analysis [51].

### 4.2. Predictors of Outcomes

There are several relevant predictors of outcomes, but especially factors related to illness onset are significant to course of illness. Statistically significant predictors of poor outcomes are presented in Table 2. Many of these factors are not modifiable, but some could possibly be affected by means of early interventions, for example, duration of untreated psychosis (DUP) [52]. One of the most important factors associated with poor outcome in schizophrenia is the early age of illness onset [27], and in the future it is necessary to further study ways to delay the onset of psychosis [53].

Clinical and functional course of illness associates also with biological markers: earlier motor development at age one [28] and morphological abnormalities in several brain areas at age 34 [29] associated with poor outcomes. The difference in early development and brain morphology in good and poor outcomes may reflect separable aetiologies and developmental trajectories in schizophrenia.

## 5. Brain Morphometry

### 5.1. Differences between Individuals with Schizophrenia and Controls

Differences between subjects with schizophrenia and controls in brain volumes and densities in several brain regions were found at the 34-year follow-up. Subjects with schizophrenia showed 2-3% lower whole brain, grey matter (GM), and white matter (WM) volumes and 7% higher cerebrospinal fluid volume when compared to controls [54]. Subjects with schizophrenia showed reduced density of GM in several brain regions, for example, frontal, temporal, parietal, and occipital regions at the 34-year follow-up. WM deficits in inter- and intrahemispheric tracts were also found in these lobes. Increased GM density was found, for example, in basal ganglia [55].

The volume of hippocampus was reduced in individuals with schizophrenia or any psychosis when compared to the controls, but the difference was explained by the total brain volume reduction. In the amygdala, significant volume differences between individuals with schizophrenia and controls were not found [56].

### 5.2. Predictors of Brain Morphometry

At age 34, associations between GM and WM densities and earlier onset age (i.e., longer duration of illness) were found [55]. One study also detected that longer DUP associated with lower GM density of the right limbic area [57]. Earlier infant motor development associated with greater density of GM in, for example, premotor cortex and WM in frontal and parietal lobes in control group, but these associations were not significant in schizophrenia [58].

Between 34 and 43 years of age, Veijola et al. [37] found the rate of annual brain volume reduction to be greater in the schizophrenia group (0.69%) than in the controls (0.49%). The greatest volume loss in schizophrenia occurred in the temporal lobe and periventricular area. The high amount of antipsychotic medication predicted excessive brain volume loss.

## 6. Cognition

Neurocognitive performance, longitudinal change in cognition, and their associations with infant motor developmental markers, brain volume change, and antipsychotic medication have all been studied in the NFBC 1966 in schizophrenia in comparison with nonpsychotic controls.

### 6.1. Developmental Markers and Cognition

Delayed infant motor development was associated with poorer adult cognition in schizophrenia at the age of 34 years in executive functions, verbal learning, and visuospatial working memory, but not in visual learning [59]. Earlier motor development, better adult executive functions, and higher grey matter density in frontocerebellar systems were associated with each other in controls, but there was a disruption of these normative associations in schizophrenia at 34 years [58].

Age of learning to stand in infancy significantly inversely predicted later deterioration of executive functions with memory in adult schizophrenia between 34 and 43 years of age [60]. This connection between delayed infant development and greater cognitive decline in adulthood may possibly suggest a link between abnormal neurodevelopmental and neurodegenerative processes in schizophrenia [60].

### 6.2. Longitudinal Change of Cognitive Performance

Global cognitive functioning as well as performance in domains of executive functions, working memory, and visual and verbal memory were lower in subjects with schizophrenia in comparison to control subjects cross-sectionally, both at the age of 34 [37, 38, 58–60] and 43 [37, 38, 60]. No significantly greater decrease in overall cognition [37] or in verbal learning [61], visual learning, or executive functions without memory component [60] was observed during midlife in schizophrenia, compared to the controls. All in all, change of cognition in schizophrenia followed mostly normative age-related decline [61]. The only exception for this pattern was the significant deterioration of executive functions with the memory component in cases compared to controls [60]. The results of mostly no deterioration extend the findings of other studies that have shown no decline in cognition around illness onset and first episode [62] also to later stages of illness in schizophrenia.

### 6.3. Antipsychotic Medication and Cognition

Higher lifetime antipsychotic medication exposure was associated with poorer verbal learning and memory performance at the age of 34 and greater decline in verbal learning and memory between the ages of 34 and 43 years [38]. Although antipsychotic exposure also predicted brain volume reduction in schizophrenia during that time, this volume reduction was not associated with a decrease in overall cognition or specific cognitive processes [37], so the associations between antipsychotic medication, cognition, and longitudinal brain changes seem complex in schizophrenia.

## 7. Somatic Illnesses, Mortality, and Criminality

### 7.1. Smoking and Somatic Illnesses

In the NFBC 1966, the proportion of smokers was about 50% among people with schizophrenia and the initiation of regular smoking was closely associated with the onset of the disorder [63]. Riala et al. [64] found poor premorbid school performance to associate with later cigarette smoking among schizophrenia patients. People with psychotic disorders have increased weight gain [65] and individuals with schizophrenia had almost a 4-fold risk of metabolic syndrome by the age of 31 [66]. The prevalence of metabolic syndrome among individuals with schizophrenia varied between 19 and 29%, depending on the measurement criteria [66, 67], and 45% had insulin resistance [68]. Total cholesterol and triglyceride levels were higher in schizophrenia compared to the controls [69], especially among those who used antipsychotic medication [40]. Also, higher triglyceride levels have been found among people with early-onset (less than 20 years of age) of schizophrenia [70]. These results imply that regular monitoring and active treatment of metabolic abnormalities are important already at a relatively young age in schizophrenia.

In addition, in the NFBC 1966, schizophrenia has been associated strongly with epilepsy by the age of 28 years, neurological diseases in general, and inflammatory bowel diseases [71], but rheumatoid arthritis and appendicitis were not more common than in the general population [72].

### 7.2. Mortality and Suicidality

In the NFBC 1966, the overall mortality rate among subjects with schizophrenia was 14% before the age of 27 years, with half of the deaths being suicides [73]. The suicide rate among people with schizophrenia before the age of 39 was 7% with 71% of suicides occurring during the first three years after the onset of illness [74]. Good school performance at the age of 16 years increased the risk of suicide in schizophrenia and other psychoses but decreased risk of suicide in the healthy controls [75]. A history of attempted and committed suicides associated with specific genetic traits among schizophrenic males [76], and the use of antipsychotic medication increased the risk of suicidal ideation among people with schizophrenia, but this association disappeared when adjusted for the severity of depression and anxiety symptoms [39].

### 7.3. Criminality and Violent Behavior

Those with psychotic disorders exhibited higher risks of criminal behavior [77], and increased risk for violent behavior was associated with schizophrenia and coexisting alcohol abuse [78] or with alcohol-induced psychoses [77]. Men with schizophrenia with comorbid alcohol abuse were also more likely to repeat the violent crimes [78]. However, also men with schizophrenia without alcohol abuse were more likely to commit violent crimes, but none of the women with schizophrenia had committed any crimes in the NFBC 1966 by the age of 26 [78].

## 8. Antipsychotic Medication

### 8.1. Use of Antipsychotic Medication

In the NFBC 1966, the associations between the use of antipsychotic medication and related factors have been extensively studied [36–40]. The predictors and consequences of the use of antipsychotic medication in schizophrenia are described in detail in Table 3. Of the predictors studied, only female gender was found to be a significant predictor of the use of antipsychotic medication.

### 8.2. Antipsychotic Medication and Outcome

The use of antipsychotic medication predicted an overall poorer outcome; for example, subjects using antipsychotics needed more hospital treatment, had poorer clinical and social outcomes, and were more often unemployed or on disability pension [36]. The subjects using antipsychotic medication had increased risk of hyperlipidemia compared to subjects not using antipsychotics [40]. Use of antipsychotic medication associated with increased suicidal ideation and symptoms of depression and anxiety [39].

### 8.3. Antipsychotic Medication, Brain Morphometry, and Cognition

Higher cumulative antipsychotic medication exposure (chlorpromazine equivalent dose-years) over the 9-year follow-up between the two brain scans was associated with brain volume reduction [37] and higher cumulative lifetime antipsychotic medication exposure associated with poorer performance and decline in verbal learning and memory in midlife in schizophrenia [38]. These associations remained statistically significant even after controlling for illness severity. Schizophrenia subjects with high antipsychotic doses declined more than the controls in some verbal learning and memory measures, whereas cognitive change in subjects with low doses did not differ from the controls [38]. The annual reduction in brain volume was significantly predicted by exposure to atypical antipsychotics but not typical antipsychotics [37], but higher exposure to both typical and atypical antipsychotics by the age of 34 associated with poorer verbal learning and memory [38].

## 9. Schizotypal Traits and Genetic Research

### 9.1. Schizotypal Traits as a Marker of Schizophrenia Risk

Schizophrenia is a highly heritable disease. Recently, the Schizophrenia Working Group of the Psychiatric Genomics Consortium [79] identified 128 independent associations spanning 108 conservatively defined loci that met genome-wide significance. The consortium also included data from the NFBC 1966. The genetic studies utilizing only the NFBC 1966 data have focused on schizotypal traits as an indication of schizophrenia risk. The schizotypal traits in the NFBC 1966 were assessed in a large (almost 5,000 individuals) follow-up at the age of 31, with the Wisconsin schizotypy scales (Social Anhedonia Scale, Physical Anhedonia Scale, and Perceptual Aberration Scale) and the Hypomanic Personality Scale.

The cohort studies have focused on psychometric properties on these scales and differences in the traits by gender and education [80]. Men reported higher anhedonia, whereas women had higher scores in hypomanic personality and perceptual aberration. Participants with a lower level of education scored higher in all scales [80]. Of the various traits, perceptual aberration and hypomanic personality associated most strongly with psychosis diagnoses in the 11-year follow-up [81], whereas physical anhedonia separated depression from other psychiatric disorders [82]. Many of the early-life risk factors linked to schizophrenia also associated with schizotypal traits, including, for instance, lower birth weight and gestational age [83].

### 9.2. Genes Associated with Schizophrenia and Schizotypal Traits

The genetic studies in the NFBC 1966 have focused especially on DISC1 (disrupted-in-schizophrenia 1), finding associations between DISC1 and social anhedonia [84]. The authors later reanalyzed the genome-wide association data and found several genes with biological relevance to mental illnesses through loci displaying suggestive evidence for association [85]. Bader et al. [86] found CRMP1 (collapsin-response mediator protein 1) locus to associate with social and physical anhedonia, suggesting the potential role of CRMP1 expression as a blood-based diagnostic marker. The NFBC 1966 data have also been used in a study that found a positive correlation between neuropeptide precursor VGF single-nucleotide polymorphisms and social anhedonia [87]. It has also been investigated as to whether large copy number variant deletions [88] or Chromosome 22q deletions [89] associate with various neurodevelopmental phenotypes. In these studies, no associations were found with regard to psychotic disorders.

## 10. Discussion

### 10.1. Main Results

The NFBC 1966 has been especially active for 20 years. The prospective study design and long follow-up have enabled several clinically and epidemiologically important findings. The early studies in the NFBC 1966 focused on risk factors for schizophrenia. Various risk factors have been found, related to, for example, family history of psychosis, adverse events during pregnancy and delivery, and delays in early developmental milestones. Psychiatric course of illness and outcomes are heterogeneous and unfortunately relatively poor. There are several predictors for outcomes, one of the most important being early age of illness onset. Some of the predictors of outcomes may be modified, such as substance use and duration of untreated psychosis.

There are differences between subjects with schizophrenia and controls in brain volume and densities in several brain areas. Global cognitive functioning and performance in various cognitive domains were worse in schizophrenia than in the controls cross-sectionally, but longitudinally cognition mostly followed the normal course of aging in midlife. High doses of antipsychotics associated with both brain volume loss and decline in cognition, and also more severe illness in terms of symptomatology, the need of hospitalization, and functional outcomes. Additionally, the use of antipsychotics was related to hyperlipidemia. Physical diseases, such as metabolic syndrome and epilepsy, are common in schizophrenia, and mortality is high, especially due to suicides.

### 10.2. Significance and Novelty of the NFBC 1966 Findings

The NFBC 1966 has provided important knowledge on epidemiology and etiology of schizophrenia. In particular, the possibility of prospectively and reliably analyzing the very early risk factors of schizophrenia is unique. Additionally, the results from the last ten years have brought information that can be utilized in developing clinical practice as well. For example, results on psychiatric and somatic outcomes, mortality, and predictors of outcomes are important with regard to the health promotion and clinical care in schizophrenia. The results also indicate that suicide prevention, early detection and intervention, monitoring somatic health, and active treatment of metabolic abnormalities are highly important. The sample is naturalistic, describing the real-life situation, so the results are generalizable to other settings. The NFBC 1966 studies have analysed also methodological questions, such as diagnostic validity [45, 46] and attrition [41–43]. The studies have been basis also for theoretical, holistic longitudinal models in schizophrenia [47].

The especially interesting and novel findings of the NFBC 1966 are those concerning potentially harmful effects of high doses of antipsychotics on changes in brain morphometry and cognitive functioning [37, 38]. These results support some earlier studies [90, 91], now detecting similar findings in unselected, nonclinical schizophrenia population. The finding of the higher cumulative lifetime dose of antipsychotics and decrease of cognition is new. Earlier studies investigating the association between antipsychotic medication and cognition in schizophrenia have been conducted most commonly in clinical trials with relatively short follow-up, in which two or more antipsychotic drugs are compared with each other. Additionally, high doses of antipsychotics were associated with increased suicidal thoughts among persons without diagnosis of psychosis (i.e., off-label use), even when depression and anxiety symptoms were controlled for [39]. Together with earlier studies [91], these results do not support the view that antipsychotics in general prevent cognitive decline or promote cognitive recovery in schizophrenia and highlight the importance of careful assessment of risks and benefits of dosage and duration of antipsychotic treatment.

### 10.3. Comparison to Other Birth Cohort Studies

The NFBC 1966 is not the only birth cohort that has studied schizophrenia. Welham et al. [1] have identified 11 birth cohort studies focusing on premorbid developmental trajectories of schizophrenia. Most consistent risk factors identified were behavioral disturbances and psychopathology, intellectual and language deficits, and early motor delays [1]. The NFBC 1966 studies have contributed to conclusions of the review, and especially results regarding early motor developmental delays are comparable to other birth cohort studies and ours. Recently, a register-based Danish National Birth Cohort study has shown that maternal HSV-2 antibodies increase the risk of schizophrenia in offspring [92]. The Prenatal Determinants of Schizophrenia (PDS) study based on the Child Health and Development Study cohort assembled in 1959–1967 in USA has identified various prenatal infection, nutrition, and toxic exposure for schizophrenia; these studies have been reviewed by Bresnahan et al. [93].

Studies on cognition on other birth cohorts have focused mostly on premorbid cognition. MacCabe [94] concluded in his review that children and adolescents with poor cognitive abilities in childhood are at increased risk of schizophrenia. Regarding adulthood cognition, patients exposed to maternal genital/reproductive infection performed more poorly on verbal memory, fine-motor coordination, and working memory in a US birth cohort (Child Health and Development Study) [95]. Also low birth weight has been linked with cognitive impairment in schizophrenia [96]. In the Dunedin cohort, including cognitive tests both before and after onset of schizophrenia disease specific decline in IQ and various mental functions in schizophrenia was found [97]. Later age of learning to stand in infancy predicted deterioration of executive functions with memory in schizophrenia between 34 and 43 years of age in the NFBC 1966 [60]. These abovementioned studies support the idea of neurodevelopmental but also neurodegenerative processes in schizophrenia.

To our knowledge only one other birth cohort study has focused on brain MRI, showing structural alterations of the cerebral cortex, particularly in the frontal and temporal lobes, and ventricular enlargement in schizophrenia [98], as in our sample.

Somatic comorbidities have been studied in the Helsinki Birth Cohort Study, where individuals with schizophrenia had a higher risk of hospitalization and mortality for coronary heart disease; however, patients with schizophrenia used less lipid-lowering and antihypertensive drug treatment [99]. These and the results of our study highlight the importance of both detecting and treating the somatic illness in schizophrenia early and efficiently. As in our study, violence and criminality have been studied, for example, in the Dunedin Birth Cohort, where the researchers found that those with schizophrenia were 2.5 times more likely to be violent than control subjects [100].

The register-based Danish national birth cohort has been utilized when studying family history and variation in single-nucleotide polymorphisms to risk of schizophrenia. Agerbo et al. [101] found that offspring schizophrenia risk was elevated in those whose mother, father, or siblings had been diagnosed with schizophrenia or related psychosis, bipolar affective disorder, or any other psychiatric disorder. Regarding schizotypal symptoms, in the Australian Mater-University of Queensland Study of Pregnancy, high childhood psychopathology predicted high levels of delusional-like experiences at the age of 21 [102].

There is also younger birth cohort based on the same geographical area than the NFBC 1966; the studies relating to schizophrenia in the Northern Finland Birth Cohort 1986 have so far studied mainly those with clinical or familial risk for psychosis or schizophrenia [103].

In all, many of the risk factor findings from the NFBC 1966 have been replicated in other birth cohorts. To our knowledge, other birth cohort studies have not analyzed clinical and occupational course of illness and their determinants after onset of schizophrenia, though these kind of clinically relevant studies would be especially important in epidemiologically sound samples like birth cohorts. There is a lack of birth cohort studies with straight clinical implications, including postmorbid data, and this should be corrected in the future as birth cohorts get older.

### 10.4. Strengths and Limitations

The strengths of the birth cohort samples in general and also of the NFBC 1966 include the prospective design and large number of unselected matched controls. This design provides unique possibilities of analyzing causal relationships, for example, between risk factors during pregnancy and delivery and later risk of illness. The major benefit in the NFBC 1966 is also the ability to utilize large nationwide health registers [104]. Linking the cohort data to register data enables researchers to study relatively easily and with low attrition various outcomes, such as hospital treatments, employment, use of social benefits, and mortality. The NFBC 1966 studies utilizing cognitive and brain imaging data differ from most of the other studies on these topics, as in those studies cases are often from clinical samples, collected from one treatment setting, and controls are not included or are selected volunteers.

The NFBC 1966 and other birth cohort studies have various limitations. Participants are not randomly assigned to exposure; thus, any observed associations may be confounded by unmeasured factors. For example, very sick individuals get more antipsychotics than persons with minimal symptomatology. Also, naturally, birth cohort studies include only case and outcome data until the point of data collection used in each study. In the birth cohort studies, the number of cases is often relatively small and power to detect even moderate signal may be low. Additionally, originally birth cohort was not planned for schizophrenia studies specifically: this means that all relevant data regarding schizophrenia research has not been collected (e.g., childhood behaviour and symptoms or treatment aspects, especially regarding psychosocial treatments).

## 11. Conclusions

Schizophrenia research has been conducted for over 20 years in the NFBC 1966. Several interesting and unique findings regarding risk factors, somatic and psychiatric outcomes, brain morphology and cognition, antipsychotics use, and genetics have been reported. In the future, with the cohort members aging, somatic health, consequences of the use of antipsychotics, and the long-term psychiatric and functional outcomes will be important to explore.

## Figures and Tables

**Table 1:** Risk factors of schizophrenia in the Northern Finland Birth Cohort 1966.

Risk factors	Risk estimates
Pregnancy and birth	
Male gender	Risk of schizophrenia OR 1.8 (95% CI 1.1–3.0) [3, 4]
Parental psychosis	Risk of schizophrenia HR 2.8 (95% CI 1.7–4.5) [5]
Maternal antenatal depression	Nonsignificant risk of schizophrenia RR 1.5 (95% CI 0.9–2.4) [6]
Unwantedness of a pregnancy	Risk of schizophrenia OR 2.4 (95% CI 1.2–4.8) [7]
High paternal social class at birth (females)	Risk of schizophrenia RR 2.4 (95% CI 1.2–4.9) [8]
Perinatal brain damage	Risk of schizophrenia OR 4.6 (95% CI 1.7–12.1) [3, 9]
Deviant intrauterine growth	Increased risk of schizophrenia for both low (OR 2.5; 95% CI 1.2–5.1) and high (OR 2.4; 95% CI 1.1–4.9) birth weight and for both low (OR 2.6; 95% CI 1.1–5.9) and high (OR 1.8; 95% CI 1.0–3.5) birth length [5, 9, 10]
Birth order	Risk of schizophrenia among male first-borns (observed-expected ratio 1.5; 95% CI 1.0–2.2) and nonsignificant risk among female last-borns (1.3; 95% CI 0.9–1.9) [11]
Grand multiparity, six or more	Nonsignificant risk of schizophrenia OR 1.1 (95% CI 0.6–2.1), significant risk of other psychosis OR 2.3 (95% CI 1.2–4.7) [12]

Childhood and adolescence	
Use of vitamin D supplementation, at least 2000 IU	Decreased risk of schizophrenia RR range: 0.08–0.23 [13]
Markers of development in childhood	Risk of schizophrenia, later age of learning to stand (RR 1.5; 95% CI 1.0–2.2) and walk (RR 1.3; 95% CI 1.0–1.6) [14]
Family type (single-parent versus two-parent) at birth to 14 years	No significant differences [15]
Viral central nervous system infection before age 14	Risk of schizophrenia OR 4.8 (95% CI 1.6–14.0) [3, 16]
Intelligence Quotient (IQ) <85 up to the age of 14 years	Risk of schizophrenia OR 4.8 (95% CI 2.2–10.3) [3]
Excellent school performance age 16 years (males)	Risk of schizophrenia OR 3.8 (95% CI 1.6–9.3) [17]
Lower school marks and below normal class age 16 years	Lower school marks associated with risk for nonpsychotic disorders, but not for schizophrenia or other psychoses. Below normal class associated with schizophrenia risk in boys OR 2.8 (95% CI 1.5–5.7) [18].

Other premorbid factors	
Severe injuries (e.g., fractures) before onset of psychosis	Risk of psychotic disorder, having a fracture HR 2.9 (95% CI 1.4–6.0) [19]
Seasonality	Rate of first admission for schizophrenia lower in spring compared to other seasons [20]
Paternal age	No significant risk by paternal age categories [21]

Interaction studies	
Parental psychosis and maternal antenatal depression	Risk of schizophrenia highest in the offspring with both maternal depressed mood during pregnancy and parental psychosis (OR 9.4; 95% CI 4.2–20.9) [22]
Parental psychosis and early risk factors	Risk for schizophrenia highest among those with parental psychosis and biological risk factor: parental psychosis and high birth weight HR 7.9 (95% CI 1.8–34.8), parental psychosis and high birth length HR 4.3 (95% CI 1.1–16.2), parental psychosis and any biological risk HR 3.6 (95% CI 1.3–10.3), and parental psychosis and high maternal education HR 0.2 (95% CI 0.1–0.9) [5]

Associations between risk factors	
Motor performance at age 1 and school performance at age 16	Age of learning to stand associated with school mark of physical education in schizophrenia but not in controls [23]
Parental psychosis and advanced paternal age	Maternal schizophrenia associated with higher advanced paternal age [24]

OR = odds ratio, CI = confidence interval, HR = hazard ratio, RR = risk ratio, and IU = international unit.

**Table 2:** Predictors of poor outcomes in schizophrenia in the Northern Finland Birth Cohort 1966.

Definition of poor outcome	Predictor of poor outcome
Poor clinical outcomes	
(i) More hospitalizations, rehospitalization after first episode [25–30]	Family history of psychosis Sociodemographic factors: father's high social class Childhood development: earlier age of learning to stand or walk Illness-related factors: insidious mode of illness onset, suicidal ideations at first episode, short first hospitalization, long DUP in short-term, and short DUP in long-term Brain morphology: reduced density of the left limbic area
(ii) More symptoms, lack of remission [27, 29, 31, 32]	Adolescence factors: smoking at age 14 years, poorer school performance at high school Illness-related factors: earlier age at onset, poor work adjustment at illness onset, single at illness onset, psychosocial stressor before illness onset, and more symptoms at first episode Temperament traits: lower reward dependence and persistence Brain morphology: decreased white matter volume
(iii) Treatment resistance [33]	Higher birth length and weight

Poor social outcomes	
(i) Poor occupational functioning (ii) Disability pension (iii) Low score on SOFAS [25, 29, 32, 34, 35]	Childhood and adolescence factors: lack of friends at childhood, poorer school performance at high school Illness-related factors: earlier age of illness onset, single at illness onset Temperament traits: lower persistence and higher harm avoidance Brain morphology: reduced density of the grey matter in left frontal lobe and left limbic area

DUP = duration of untreated psychosis, SOFAS = Social and Occupational Functioning Assessment Scale.

**Table 3:** Factors associated with the use of antipsychotic medication in the Northern Finland Birth Cohort 1966.

Factors	Effect of higher use of antipsychotic medication
Sex	Females used more often medication [36]
Age at onset of psychosis	No association [36]
Psychiatric hospital treatment	Increased need of hospital treatment [36]
Clinical measures (PANSS, CGI, SOFAS, and remission)	Poorer clinical and social outcome [36]
Occupational functioning	Higher rate of disability pensions and lower rate of employment [36]
Brain volume loss	Increased brain loss [37]
Change of cognition	Decline of verbal learning and memory [38]
Suicidal ideation	No association when symptoms of depression and anxiety were controlled for [39]
Hyperlipidemia	Increased risk of hyperlipidemia [40]

PANSS = Positive and Negative Syndrome Scale, CGI = Clinical Global Impression, and SOFAS = Social and Occupational Functioning Assessment Scale.
